# Sequence-specific detection of single-stranded DNA with a gold nanoparticle-protein nanopore approach

**DOI:** 10.1038/s41598-020-68258-x

**Published:** 2020-07-09

**Authors:** Loredana Mereuta, Alina Asandei, Isabela S. Dragomir, Ioana C. Bucataru, Jonggwan Park, Chang Ho Seo, Yoonkyung Park, Tudor Luchian

**Affiliations:** 1 Department of Physics, ‘Alexandru I. Cuza’ University, 700506 Iasi, Romania; 2 Sciences Department, Interdisciplinary Research Institute, ‘Alexandru I. Cuza’ University, 700506 Iasi, Romania; 3 Department of Bioinformatics, Kongju National University, Kongju, 32588 Republic of Korea; 4 Department of Biomedical Science and Research Center for Proteinaceous Materials (RCPM), Chosun University, Gwangju, 61452 Republic of Korea

**Keywords:** Molecular biophysics, Nanoscale biophysics, Single-molecule biophysics

## Abstract

Fast, cheap and easy to use nucleic acids detection methods are crucial to mitigate adverse impacts caused by various pathogens, and are essential in forensic investigations, food safety monitoring or evolution of infectious diseases. We report here a method based on the α-hemolysin (α-HL) nanopore, working in conjunction to unmodified citrate anion-coated gold nanoparticles (AuNPs), to detect nanomolar concentrations of short single-stranded DNA sequences (ssDNA). The core idea was to use charge neutral peptide nucleic acids (PNA) as hybridization probe for complementary target ssDNAs, and monitor at the single-particle level the PNA-induced aggregation propensity AuNPs during PNA–DNA duplexes formation, by recording ionic current blockades signature of AuNP–α-HL interactions. This approach offers advantages including: (1) a simple to operate platform, producing clear-cut readout signals based on distinct size differences of PNA-induced AuNPs aggregates, in relation to the presence in solution of complementary ssDNAs to the PNA fragments (2) sensitive and selective detection of target ssDNAs (3) specific ssDNA detection in the presence of interference DNA, without sample labeling or signal amplification. The powerful synergy of protein nanopore-based nanoparticle detection and specific PNA–DNA hybridization introduces a new strategy for nucleic acids biosensing with short detection time and label-free operation.

## Introduction

Assay designs effective in diagnostic workflow for early detection of pathogen outbreaks, as the recently emerged novel coronavirus disease 2019 (COVID-19), pose serious challenges even for specialized research or public health laboratories, especially in low-income countries. Besides culture and colony counting, or immunological investigations, hybridization assays built around polymerase chain reaction (PCR)-based strategies represent the golden standard when it comes to quantification of disease-specific nucleic acids. The emergence of new technologies such as real time PCR, microfluidics, integrated rapid PCR systems, and massively parallel sequencing devices, facilitated the introduction of new methods enabling effective disease detection. Comprehensive information regarding pros and cons of these methods has been presented elsewhere^1–4^. Despite successes, probing the presence of target sequences carried by exogenous nucleic acids by traditional hybridization assays^5,6^, suffers from serious limitations generated by nonspecific amplification or hybridization, as well as costly infrastructure and complex work. To mitigate such challenges, nanomaterials (e.g., quantum dots, carbon nanotubes) were employed for fluorescence assays of nucleic acids^7–10^ while others demonstrated the ability of polyelectrolyte-modified capacitive EIS sensors for the label-free detection of DNA hybridization in aqueous buffers^11,12^ or silicon nanowire-based field-effect transistors^13^. Single-molecule, fluorescence resonance energy transfer and fluorescence correlation spectroscopy methods, were also used to detect duplex formation within or between individual DNA strands^14–18^.

Gold nanoparticles (AuNPs) emerged as nanomaterials with a game-changing potential in the field of nucleic acids detection^19^. Central for traditional colorimetric assays, the crucial observation is that the peak absorbance wavelength of AuNPs is sensitive to the distance between particles. If a target analyte triggers AuNP aggregation, the surface plasmon resonance of individual AuNP particles becomes coupled and shifts the absorbance spectrum, as non-aggregated nanoparticles present a red color, while aggregated nanoparticles appear blue^20^. The first AuNP-based DNA sensor was developed by Mirkin and co-workers, whereby they modified two sets of AuNPs with different single-stranded DNA probes and mixed them with a target DNA. If the target DNA presents sequences complementary to both the probes, it will cause the particles to cross-link by hybridization and aggregate^21^. In another seminal study, only one kind of single-stranded probe DNA was grafted on AuNPs, and it was demonstrated that in the presence of perfectly complementary target DNA, AuNPs aggregated via non-cross-linking DNA hybridization^22^. Others revealed that single- and double-stranded oligonucleotides have different electrostatic properties to protect AuNPs from salt induced aggregation, that can be used for DNA detection without covalent immobilization of DNA onto AuNPs^23–27^. By means of spectroscopy, quasielastic light scattering or isothermal titration calorimetry, interactions between AuNPs and nucleobases^28^ or mononucleotides and oligonucleotides^24,29–31^ have been characterized in great detail. In a recent review, the applications of gold nanoparticles-based assays as viable alternatives to conventional methods, for detection of infectious diseases, are thoroughly discussed^32^.

We sought herein to find a solution for future development of biosensors enabling rapid detection of exogenous ssDNA with high sensitivity, in a cost-effective way. Unlike the colorimetric or spectroscopic assays reviewed before, we resorted to the utilization of a homo-heptameric α-hemolysin (α-HL) protein nanopore^33^ working in conjunction with AuNPs and a particular class of artificial genetic polymers, as an approach capable of rapid discrimination of short ssDNAs. Built on a strategy similar to the original Coulter-like counter^34–36^, nanopores emerged as versatile systems for detecting small molecules^37–41^, pathogens^42,43^, RNA and DNA^44–49^ or peptides and proteins^50–55^.

With relevance to detecting nucleic acid targets via hybridization-assisted nanopore sensing, peptide nucleic acids (PNAs)^56^ constitute promising reagents due to their resistance to enzymatic degradation by nucleases and proteases^57^ and strong hybridization properties with complementary DNA/RNA targets^58^. The monitoring of PNA-based hybridization with nanopores was shown to be particularly useful for selective miRNA^59^ or ds/ssDNA detection^60–66^. In particular, the involvement of PNA in AuNPs-based nanoassembly and assays for DNA detection was reported in pioneering studies^67–70^. Only a handful of previous studies investigated the utility of α-HL as a sensitive platform for characterizing water-soluble gold nanoclusters with different ligand shell composition^71–74^, whereas others have involved pores manufactured in polymeric membranes in conjunction with AuNPs to detect DNA targets^75^.

The strategy at the core of this work was to use resistive pulse sensing through a single α-HL nanopore, to sense and discriminate AuNPs based on their specific PNA-induced surface properties changes and aggregation propensity, closely related to the PNA-target ssDNA hybridization. By virtue of its scalability to a wide range of ssDNA sequences, cost-effectivity, high sensitivity and specificity, this method illustrates a potentially solid alternative for amplification-free, hybridization-based exogenous nucleic acids detection in the field, biotechnological applications or point-of-care units.

## Materials and methods

### Reagents

The custom designed peptide nucleic acids (PNA2) were synthesized by and purchased from Panagene Inc., South Korea, and the complementary (HCV) or noncomplementary (H1N1) ssDNAs sequences to PNA2 were purchased from Sigma-Aldrich, Germany (see Table 1). Citrate-coated gold nanoparticles (AuNPs) with a diameter ~ 5 nm, human serum (HS) and other reagents including potassium chloride (KCl), sodium chloride (NaCl), ultra-pure water (DNAase and RNAase free), EDTA, Tris buffer, *n*-pentane, hexadecane, dimethyl sulfoxide (DMSO) and α-hemolysin (α-HL), were purchased from Sigma–Aldrich, Germany. The 1,2-diphytanoyl-sn-glycerophosphocholine (DPhPC) lipids were obtained from Avanti Polar Lipids, Alabaster, AL, USA.

**Table 1:** Primary sequence and the molecular weight of the PNA and ssDNA fragments used in this work.

Polynucleotide	Primary sequence	Mw (g/mol)
PNA2	5′-CCCACCCGCAGCCCTCATTA-3′	5,263.1
HCV	5′-TAATGAGGGCTGCGGGTGGG-3′	6,297
H1N1	5′-ACG GAAGGA GTGCCAA-3′	4,968

In accord to the accepted convention, the N-terminal of the PNA2 is referred to as its 5′-end.

### Buffer solutions

Depending on the experiment, electrolyte solutions for UV–vis spectral analysis or electrophysiology contained various amounts of salt (0.1 M or 3 M KCl), 1 mM EDTA, buffered in 10 mM Tris at pH = 7.3. All experiments were performed at room temperature ~ 23 °C.

### Sample preparation

The dried form of ssDNAs samples and PNA2 were dissolved in 1 M NaCl solution in ultra-pure water buffered with TE (10 mM Tris, 1 mM EDTA) at pH = 8.2, to obtain stock solutions of 100 μM and 5 μM concentration, respectively. In order to enhance the solubility of PNA2, small amounts of DMSO (5% v/v) were added in the stock solution. After solvation, all samples were vigorously stirred using a Stuart BioCote vortex mixer (Sigma–Aldrich, Germany) at 1,400 rpm, for 3 min, in continuous mode. To improve rehydration, the samples were heated up to 95 °C for 20 min using an IKA Digital Block Heater (Cole-Parmer, USA) and slowly cooled down to 23 °C. All stock solutions were divided in aliquots and kept at − 20 °C. Prior use on specific experiment, HCV, H1N1 or PNA2 aliquots were annealed separately by rapidly heating each sequence to 95 °C and slowly cooling to 22 °C, using an IKA Digital Block Heater (Cole-Parmer, USA). Mixtures of PNA2–HCV or PNA2–H1N1 at 1:4 concentration ratio, were formed by incubation of specific sequences for 30 min at room temperature, in 1 M NaCl solution made in ultra-pure water buffered with TE (10 mM Tris, 1 mM EDTA) at pH = 8.2. The AuNP solution was kept at 4 °C and brought to room temperature before specific measurements.

### Spectral analysis of PNA- and ssDNAs-induced aggregation of AuNPs

To investigate the stability of AuNPs samples and the PNAs- and ssDNAs-induced aggregation of AuNPs, UV–vis spectroscopy experiments were performed using a NanoDrop OneC spectrophotometer (Thermo Fisher Scientific, USA). In such experiments, 950 µL of reference solution (0.1 M KCl buffered with 1 mM EDTA and 10 mM Tris, or 3 M KCl buffered with 1 mM EDTA and 10 mM Tris around pH = 7) was pipetted in a quartz cuvette with a 10-mm path length, and the absorption spectra were recorded in the 400–800 nm wavelength range. Subsequently, 50 µL from a AuNP stock solution (100 nM) were added, to attain a final concentration of 5 nM AuNP in the cuvette, and absorption spectra were recorded in the similar wavelength range. An extinction coefficient of 8.56 × 10^6^ L/(mol cm) was used to verify the AuNPs concentration in solution. Depending on the experimental protocol, HCV, H1N1, pre-mixed PNA2–HCV or PNA2–H1N1 sequences were added at required concentrations in the quartz cuvette, and the spectral shift of the surface plasmon peak of the AuNP solution was measured. When needed, the experiments were repeated in similar electrolytes, but containing of 10% or 3% human serum.

### Nanopore electrophysiology

The electrophysiology experiments were performed in a recording chamber consisting of two compartments denoted by *cis* (grounded) and *trans*, separated by a 25 µm-thick Teflon film (Goodfellow, Malvern, MA, USA), containing an aperture of about 120 µm in diameter. The lipid bilayer was obtained from 1,2-diphytanoyl-sn-glycero-phosphocholine dissolved in n-pentane, HPLC-grade (10 mg/mL), across the aperture in the Teflon film, pre-treated with a mixture of 1:10 hexadecane in n-pentane^64^. Importantly, to prevent salt-induced AuNPs aggregation, the *cis* compartment containing AuNPs was filled with 0.1 M KCl solution buffered with 1 mM EDTA, 10 mM Tris, at pH = 7.3, whereas the *trans* compartment contained 3 M KCl in an otherwise similar buffer. After a stable lipid membrane was obtained, ~ 0.5 to 2 µL α-HL from a monomeric stock solution made in 0.5 M KCl were added to the grounded, *cis-*compartment. After ~ 15 min under continuous stirring, a single heptameric α-HL nanopore formed into the lipid membrane. Prior to applying the desired holding voltage across the lipid membrane, the residual transmembrane potential across the α-HL, generated due its slight anionic selectivity at neutral pH, was nullified with the voltage compensation knob on the current amplifier. Subsequently and depending on the experiment, AuNP, PNA2, HCV and H1N1 (alone or pre-mixed) were added at the desired concentrations on the *cis* side of the nanopore. Current fluctuations reflecting AuNPs–α-HL interactions in the absence or presence of nucleic acids were recorded in the voltage-clamp mode via two Ag/AgCl electrodes connected to an Axopatch 200B or Multiclamp 700B amplifier (Molecular Devices, USA). The electrical signals were digitized at a sampling frequency of 50 kHz with a NI PCI 6,221 16-bit acquisition board (National Instruments, USA) and low-pass filtered at 10 kHz. In order to reduce the effect of environmental noise, the recording system was shielded in a Faraday cage (Warner Instruments, USA), and placed on a vibration-free platform (BenchMate 2210, Warner Instruments, USA). To facilitate the control and recording of the electrical signals, a virtual instrument was developed within LabVIEW 8.20 platform (National Instruments, USA). The analysis of the ionic current blockades across the α-HL was performed within the statistics of exponentially distributed events, as previously described^64^. All numerical analysis and graphic representations of the recorded data were done using pClamp 6.03 (Axon Instruments, USA) and Origin 6 (Origin Lab, USA).

## Results and discussion

### Principle of the experiment

Knowing that in certain cases effective screening for viral infection requires detecting short ssDNA sequences in biological fluids (e.g., blood serum), which constitute biomarkers for the pathogen’s presence^60^, we proposed herein a strategy to detect a sequence-specific single-stranded DNA (named herein HCV) particular to the infection with hepatitis C virus^76^. For the negative control experiment, we used another short ssDNA sequence, constituting a biomarker for the H1N1 virus presence^77^. In Table 1 we present the ssDNA sequences used herein.

**Figure 1:**
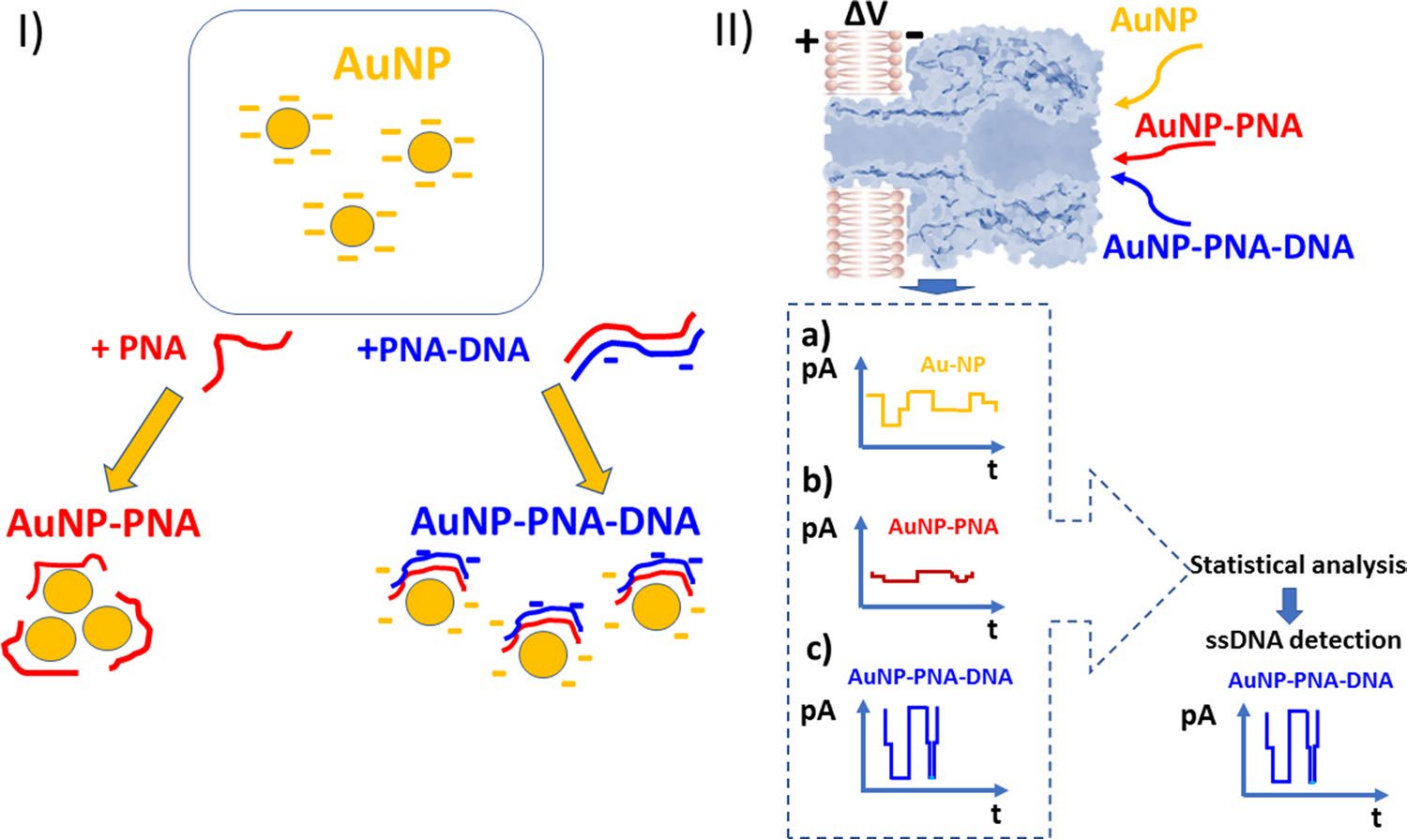
Schematics of α-HL-mediated ssDNA detection via PNA-induced AuNP aggregation. (**I**) AuNPs aggregate in the presence of PNA, and subsequent addition of the complementary ssDNA sequence disrupts the particle aggregation significantly. (**II**) Each state illustrated in panel I can be discerned via single-particle measurements of α-HL–AuNP interactions and specific blockade signature (**a**—AuNP alone; **b**—AuNP–PNA aggregates; **c**—ssDNA-induced disruption of AuNP–PNA aggregates), thus facilitating ssDNA detection.

In Fig. 1 we lay out the basic idea underlying the use of the citrate ions-protected AuNPs–α-HL system, as a potentially useful nanopore platform to detect distinct signals suggestive of the ssDNA presence in electrolyte, via selective hybridization with complementary PNAs (named herein PNA2, see Table 1).

As seen, in the presence of charge-neutral PNAs, citrate ions-protected AuNPs undergo immediate aggregation, since the adsorption of PNA shields weakly bound citrate ions, causing the loss of charge repulsion^70^. Based upon their size, such assemblies are readily discernable from dispersed citrate ions-protected AuNPs, through the distinct blockade signature entailed on the ionic current measured across a single α-HL nanopore. We anticipated that as the presence of complementary ssDNA in solution will disrupt the particle aggregation significantly, this become readily visible as a distinct blockade signature on the α-HL, and thus serve as a facile and rapid detection system for the presence of the complementary ssDNA.

### UV–vis spectroscopy analysis of the PNA- and ssDNAs-induced aggregation effect on gold nanoparticles

**Figure 2:**
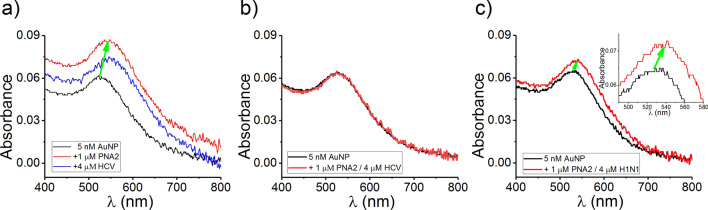
UV–Vis absorption spectral data on PNA- and ssDNA-induced aggregation of AuNP. (**a**) Addition of 1 µM PNA2 in a 0.1 M KCl containing 5 nM AuNP, determines the spectral shift of the surface plasmon peak (A_max_) from 520 nm and A_max_ = 0.06 to 550 nm and A_max_ = 0.086 (5 min of incubation time; red line). The spectral shift was not reversible after addition of 4 µM from the complementary ssDNA HCV and incubation of approximately 20 min (the surface plasmon peak (A_max_) = 550 nm and A_max_ = 0.073; blue line). (**b**) When preformed PNA2–HCV complexes were added on the 0.1 M KCl electrolyte containing 5 nM AuNP, no spectral shift was observed after 5 min incubation time. (**c**) If 1 µM PNA2 is premixed with 4 µM from the noncomplementary ssDNA H1N1 in the AuNP solution (5 nM), a shift of the surface plasmon peak is visible after 5 min of incubation from 525 nm and A_max_ = 0.064 to 535 nm and A_max_ = 0.072.

In a first series of experiments aimed at rapid, macroscopic verification of the paradigm depicted above, we investigated the PNA2-induced AuNPs aggregation with the involvement of PNA2 oligomers, in the absence and presence of complementary ssDNA (HCV). Figure 2, panel a, shows the UV–vis absorption spectrum of the citrate-stabilized AuNPs (5 nM) dispersed in a buffer containing 0.1 M KCl, with the maximum absorption wavelength observed around 520 nm. The concentration of the AuNPs were calculated using Beer’s law and extinction coefficient for 5 nm AuNPs (8.56 × 10^6 ^L/(mol cm)^78^. We sought to find a working compromise for setting the optimal salt concentration in the AuNP-containing buffer, as to mitigate possible salt-induced, electrostatic stabilization and aggregation of citrate-caped AuNPs^79^ and at the same time improve selectivity to detect target ssDNA^70^, and achieve experimental conditions amenable to subsequent electrophysiology experiments. As we demonstrate in Supplementary Fig. S1, when incubated for ~ 30 min in a 0.1 M KCl containing buffer, the AuNPs remain in a stable dispersed state. This is reflected by the lack of shift of surface plasmon peak from 520 nm to longer wavelengths.

When AuNPs were mixed with the PNA2 sequence (1 µM), the surface plasmon peak shifted from 520 to 550 nm, indicating that the PNA2 molecules occupied the citrate binding sites leading to the loss of surface negative charge and AuNP aggregate formation in solution^68,69^. Interestingly and contrary to our expectations, subsequent addition in excess of the complementary HCV ssDNA (4 µM) did not recover the shift in the surface plasmon peak caused by PNA2 adsorption to the AuNPs. We anticipated that PNA2–HCV hybridization taking place on the surface of AuNPs previously interacted with PNA2 would impart a net negative surface charge on the nanoparticles and protect them against PNA2-induced aggregation. In contrast, addition of pre-hybridized PNA2–HCV complexes prevented AuNPs aggregation (Fig. 2b). This is explainable through the electrostatic repulsion interactions manifested among PNA2–HCV–AuNPs, due to the negative phosphate backbone of the DNA strands from the PNA2–HCV complexes adsorbed onto the AuNPs surface.

To further validate such findings, we carried out distinct experiments during which PNA2 was pre-incubated with the non-complementary H1N1 ssDNA sequence, and the mixture was subsequently added in the electrolyte solution containing dispersed AuNPs. Data shown in Fig. 2c, demonstrate the emergence of a shift in the surface plasmon peak, attributable to the non-hybridized PNA2 molecules which bind to the AuNPs and induce aggregation. Although the non-hybridized H1N1 ssDNA fragments were expected to stabilize AuNPs against aggregation^23^, their failure to compete with PNA2 leading to PNA2-induced aggregation, strengthens the fact that due to the particular backbone properties of PNA2, they bind dominantly to AuNPs relative to ssDNA^70^.

To further test for the interplay of AuNPs with serum proteins, as an early indicator of the method’s performance detection target ssDNAs in a biological system, we extended our experiments to the case when the electrolyte solution contained human serum as a mimic to clinically-relevant samples. As known, AuNPs interaction with various proteins found in the bloodstream, generate the emergence of the protein layer on the nanoparticle surface, named protein corona^80^, which influences the conformation and surface properties of nanoparticles. As shown in Supplementary Fig. S2, incremental addition of 3% and 10% of human serum of a 0.1 M KCl electrolyte led to a gradual decrease in the absorbance of surface plasmon peak accompanied by a shift of surface plasmon peak to longer wavelengths, all indicative of proteins adsorption on the AuNPs surface and concomitant changes in their aggregation propensity^81^. Further, successive addition of PNA2 (1 µM) and HCV (4 µM) fragments in a solution containing AuNP (5 nM), and presence of 10% human serum, led to a gradual decrease in the absorbance of the surface plasmon peak accompanied by a negligible shift of its wavelength (Supplementary Fig. S3). These data demonstrate that hurdles lie ahead in trying to unambiguously extrapolate the findings above, to employ AuNPs and PNAs for detecting complementary ssDNA strands in clinical samples.

### Nanopore detection of ssDNA fragments with the AuNP/α-HL platform

Having established the feasibility of the HCV detection with the PNA2–AuNP system, we next focused on testing the α-HL protein-based nanopore platform, as a candidate to facilitate selective detection of HCV in real-time. Given the ~ 2.6 nm diameter at the α-HL’s vestibule entry^33^, in all experiments we added the nanoparticles from the *cis* chamber. In doing so, we sought to facilitate optimal capture events of AuNPs at the nanopore’s vestibule entry, easily discernible through reversible blockades of the ionic current across it. *As* *a* *crucial* *step,* *in* *all* *experiments* *we* *maintained* *salt* *gradients* *across* *the* *nanopore* *(trans* *3* *M* *KCl/cis* *0.1* *M* *KCl),* as this impacted two major advantages on the platform: (1) established an optimal salt concentration in the *cis* compartment where AuNP, HCV and PNA were allowed to interact, in order to minimize the likelihood of salt-induced AuNP aggregation, and (2) increased both the AuNP capture rate from the *cis* bulk and signal-to-noise ratio of the blockade events^82^.

**Figure 3:**
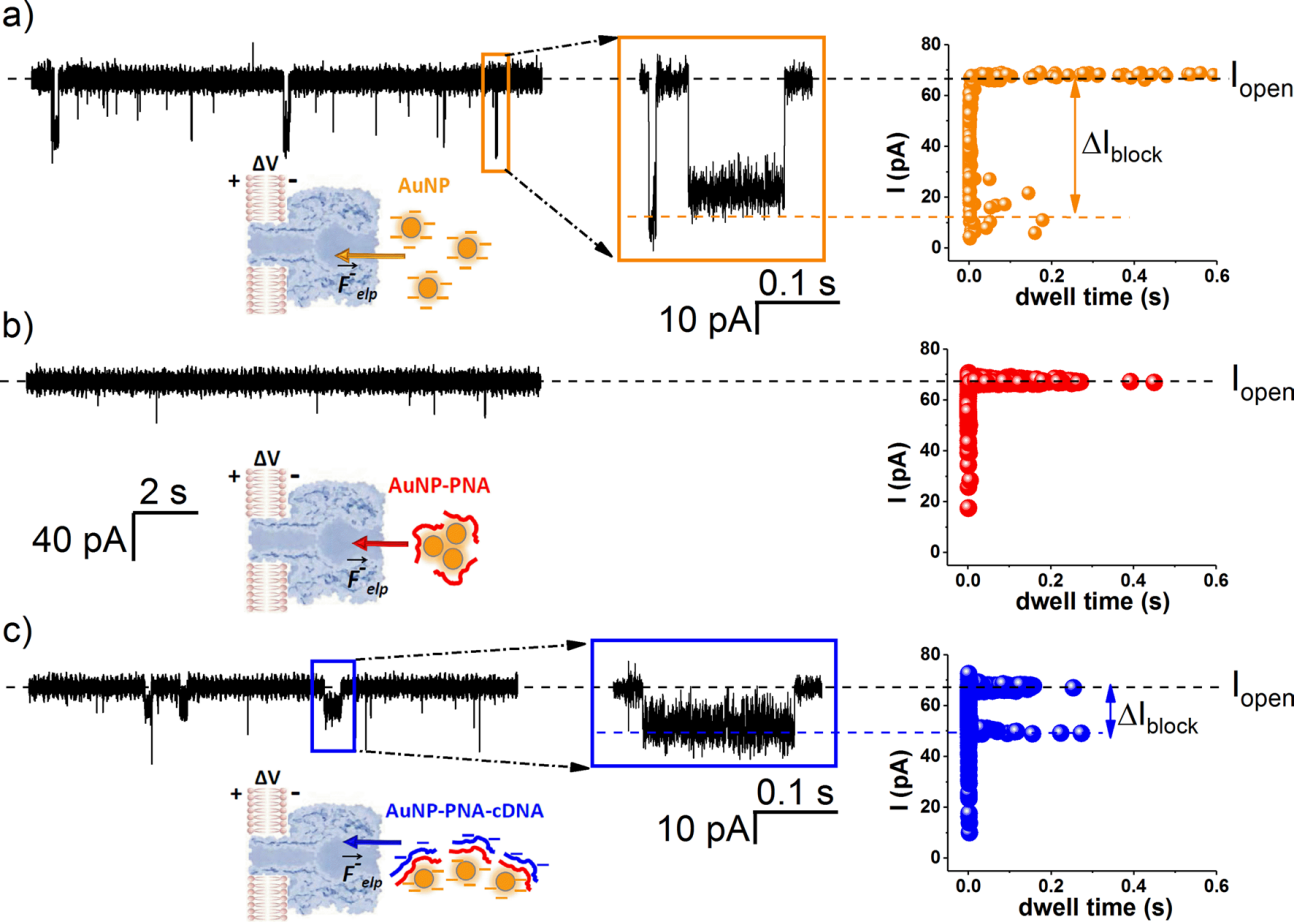
Detection of ssDNA fragments with AuNPs on a α-HL nanopore platform. (**a**) Single AuNP fingerprinting characterizing ionic currents blockades induced by AuNP–α-HL nanopore interactions. AuNP (5 nM) were added on the *cis* side of the nanopore subjected to a transmembrane potential of ΔV =  + 70 mV, bathed in asymmetric salt buffers (*trans* 3 M KCl/*cis* 0.1 M KCl), at neutral pH. The scatter plot reveals that AuNP-induced blockade on the free ionic current (I_open_) through the nanopore equals ΔI_AuNP_ = I_open _− I_block_ = 48.1 ± 0.8 pA and a mean blockade duration of τ_off_AuNP_ = 0.001 ± 5E − 4 s. (**b**) Subsequent *cis*-side addition of PNA2 (1 µM) leads to a practically instantaneous cessation of blockade events reflecting AuNP–α-HL nanopore interactions, as seen in a, and the associated scatter plot illustrates this further. (**c**) The *cis*-side addition from the complementary HCV (4 µM) restores the AuNP-induced blockade activity on the nanopore. Remarkably, and as illustrated by the scatter plot, this blockade signature is characterized by shallower (ΔI_Au/PNA2/HCV_ = 15.4 ± 0.39 pA) and longer lasting blockade substates (τ_off_Au/PNA2/HCV_ = 0.12 ± 0.013 s), as compared to those recorded in (**a**). The orange and respectively blue rectangles in (**a**) and (**c**), highlight blockade events under analysis.

Selected electrophysiology traces displayed in Fig. 3 capture the chain of events seen in UV–vis absorption spectra (Fig. 2), but with an increased sensitivity, enabling single nanoparticles monitoring. Due to the net negative charge on the citrate-coated nanoparticles surface^70^, *cis*-side added AuNPs (5 nM) are reversibly captured at vestibule opening of the nanopore subjected to a *trans* positive potential, and the process is seen as stochastic reductions of the ionic current flowing through the open α-HL (Fig. 3a). As shown in Supplementary Fig. S4a, increased transmembrane potentials (ΔV) across the nanopore led to longer dissociation times (τ_off_) of the AuNP from the nanopore. This clearly makes sense, as negatively charged AuNP move against the electric field during dissociation from the nanopore, so that the AuNP–α-HL dissociation process is energetically unfavored.

Subsequent *cis*-side addition of PNA2 (1 µM) resulted in an almost complete cessation of the AuNP-induced blockade events on the nanopore current (Fig. 3b). This stems most likely from the nanoparticle aggregation and formation of larger sized conjugates, induced by PNA2 adsorption onto their surface (vide supra), leading to clusters whose dimensions exceed by far the opening diameter of the α-HL’s vestibule. These clusters are precluded from successful capture at the nanopore entrance, and are likely contributors for the emergence of some spurious, very short lasting, bumping-like events (see also Fig. 5b, e). The sterically-based exclusion of AuNP aggregates capture by the nanopore is supplementary supported by the fact that the overall charge on the PNA2-coated AuNPs remain largely negative^70^, meaning that PNA2-induced AuNP aggregates are still prone to the electrophoretically-driven transport toward the nanopore’s vestibule, by *trans*-positive potentials. Addition of the complementary HCV sequence (4 µM) (Fig. 3c) was seen to generate milliseconds-long blockade events with a reduced amplitude as compared to those presented in panel a. Having established that under such circumstances, complementary HCV addition does not re-disperse PNA2-induced AuNP aggregates (Fig. 1a), it is still debatable the origin and structure of aggregates responsible for this blockade fingerprint (blue rectangle in Fig. 3c), not seen by conventional UV–vis methods. It was unlikely that the free HCV strand could have led to the emergence of such events, as α-HL interaction with comparably short ssDNA sequences are sub-milliseconds long^66^ (Supplementary Fig. S4b).

To further test the feasibility of the method, in a next set of experiments we probed the interactions between the α-HL nanopore and the dispersed AuNPs in interaction with pre-formed PNA2–HCV duplexes, obtained following incubation of PNA2 and HCV.

**Figure 4:**
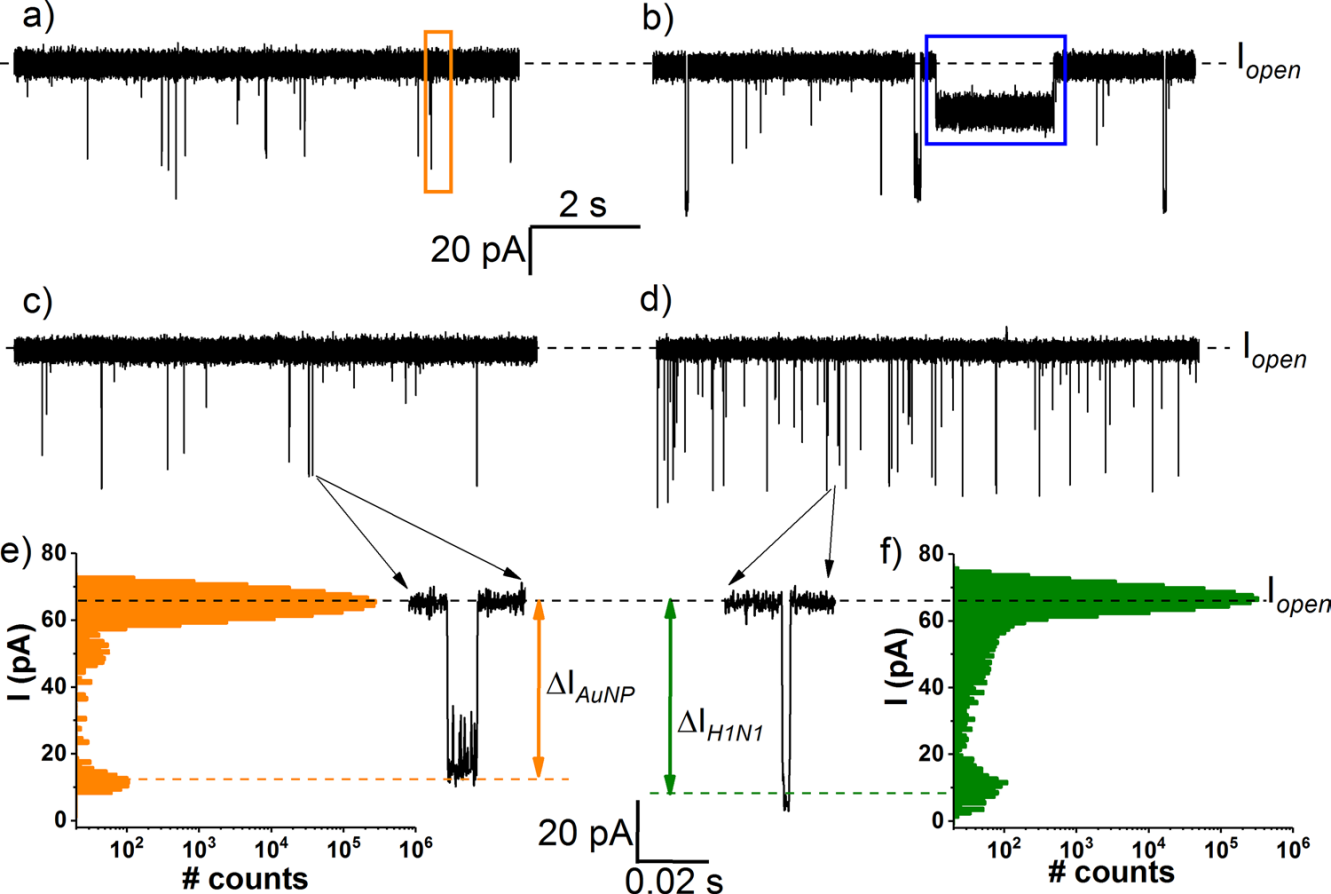
Selective detection of the ssDNA target complementary to the PNA probe, with the AuNP/α-HL platform. (**a**, **c**) Blockade events (ΔI_AuNP_ = 57 ± 1.2 pA; τ_off_AuNP_ = 0.002 ± 9.5E−4 s) characterizing *cis*-side added AuNP (5 nM)–α-HL nanopore interactions, at ΔV =  + 80 mV, recorded in asymmetric salt buffers (*trans* 3 M KCl/*cis* 0.1 M KCl) at neutral pH. (**b**) Subsequent *cis*-side addition of preformed PNA2 (1 µM)–HCV (4 µM) complexes led to a similar type of blockade events as recorded when PNA2 and HCV were added successively (Fig. 3, (**c**), indicating HCV detection. (**d**) *Cis*-side addition of the mixture containing preincubated noncomplementary H1N1 (4 µM) and PNA2 (1 µM) fragments resulted in blockade events which, based on their amplitude (ΔI_H1N1_ = 60.25 ± 0.91 pA) and duration (τ_off_H1N1_ = 0.0004 ± 5.6E−5 s), were indicative of free, ssDNA H1N1 interaction with and translocation across the α-HL (see also Supplementary Fig. S5).

As shown in Fig. 4, two distinctive levels of current blockades were observed: (1) events similar to those generated by AuNP–α-HL interactions (Fig. 3a), highlighted in Fig. 4a, by the orange rectangle; (2) events of a reduced amplitude and longer-lasting duration (Fig. 4b, blue rectangle). Because the current change magnitude through the nanopore is closely related to the excluded volume of the molecule blocking the nanopore, the ionic signatures of events depicted as blue rectangle in Fig. 4b—which resemble those seen when PNA2 and HCV were added sequentially (Fig. 3c)—were attributed to the specific, distinct population of AuNP/PNA2–HCV nanoparticles.

To confirm that current transients registered in Fig. 4b, blue rectangle, were indicative of the specific PNA2–HCV hybridization, we performed a negative control by adding the pre-incubated PNA2–H1N1 to the AuNP-containing electrolyte. As shown in Fig. 4d, this resulted in current blockade events with a signature close to those recorded during freely added H1N1 interactions with α-HL (ΔI_*H1N1*_ = 52 ± 1.6 pA and τ_off*_H1N1*_ = 0.00014 ± 1.7E−5 s; Supplementary Fig. S5a) or freely added HCV interactions with α-HL (ΔI_*HCV*_ = 61.8 ± 0.53 pA and τ_off*_HCV*_ = 0.0012 ± 1.48E−4 s; Supplementary Fig. S5b).

To interpret this, we recall that due to their sequence mismatch, PNA2 and H1N1 fragments will not hybridize. Therefore, upon addition of the pre-incubated PNA2–H1N1 in the *cis* chamber, already containing dispersed AuNPs, the free, non-hybridized PNA2 component will promote AuNPs aggregation, and the resulting conglomerates will be excluded from capture at the nanopore’s vestibule entry due to their large excluded volume (vide supra, Fig. 3b). On the other hand, the non-hybridized H1N1 ssDNA fragments will interact with and be transported across the α-HL nanopore, leading to the fast occurring blockade events shown in Fig. 4d. The significant differences between the blockade signatures on ionic current–time traces reflecting the reversible interaction of a single α-HL nanopore with AuNP/PNA–HCV or AuNP/PNA–H1N1 complexes, verified the successful discrimination of the HCV fragments in electrolyte.

### Exploring the sensitivity limit of selective target ssDNA detection with the AuNP/α-HL platform

**Figure 5:**
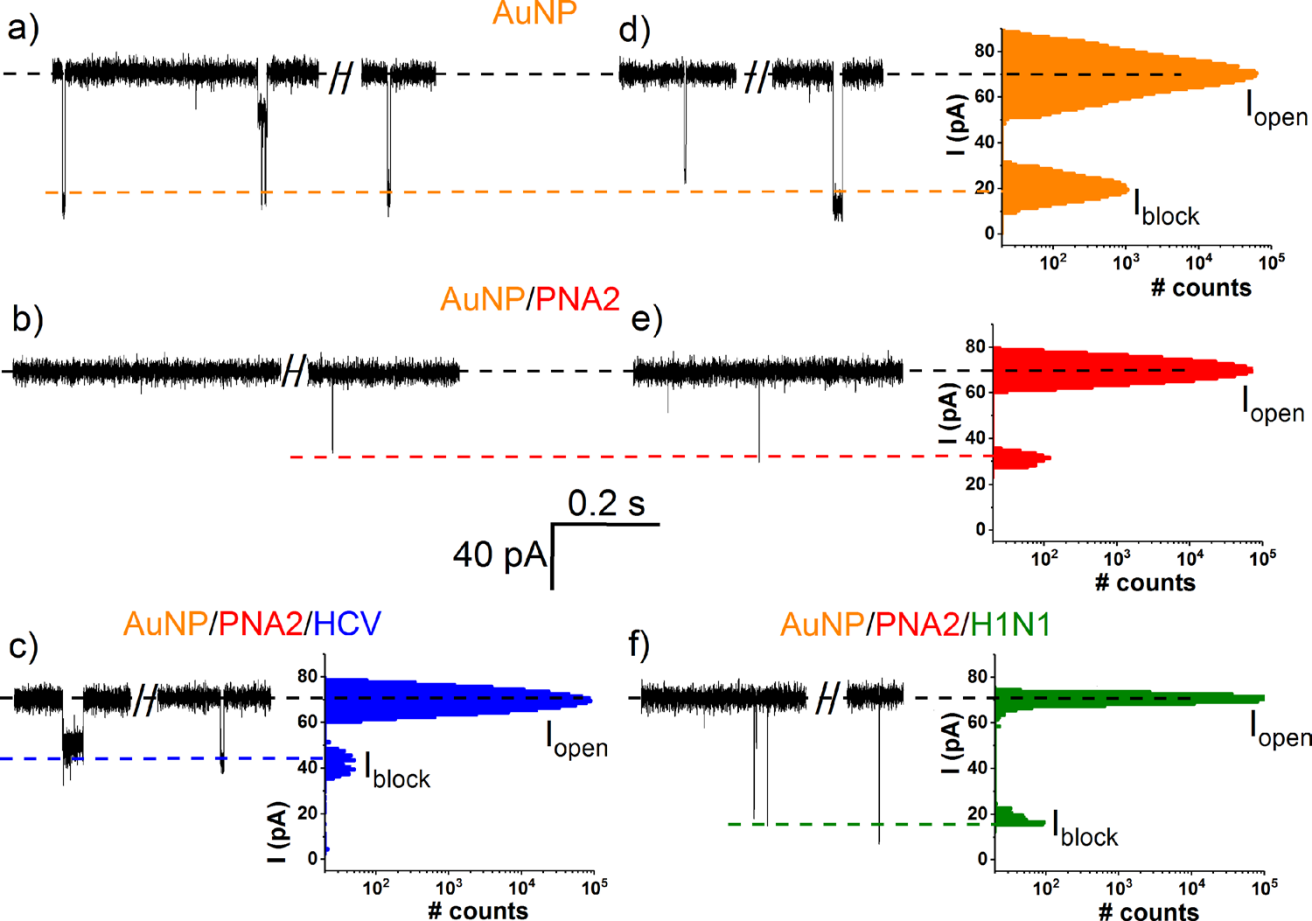
Specific detection of HCV fragments detection with the AuNP/α-HL platform at nanomolar concentration. Excerpted ionic current traces recorded in separate experiments through the α-HL (ΔV =  + 80 mV) and corresponding all-points histograms, after successive *cis* side addition of 5 nM AuNP (ΔI_AuNP_ = 57 ± 1.2 pA; τ_off_AuNP_ = 0.002 ± 9.5E−4 s) (**a**, **d**), 5 nM PNA2 (**b**, **e**), 5 nM HCV (ΔI_Au/PNA2/HCV_ = 20.5 ± 0.3 pA; τ_off_Au/PNA2/HCV_ = 0.72 ± 0.07 s) (**c**) or 5 nM H1N1 (ΔI_H1N1_ = 61.7 ± 0.9 pA and τ_off_H1N1_ = 0.0002 ± 4.1E−5 s) (**f**).

To further test the sensitivity and selectivity of HCV detection, similar experiments as in Fig. 3 were carried out, except that PNA2 (5 nM) and either HCV (5 nM) or H1N1 (5 nM) were added in the *cis* side of the recording chamber, already containing AuNPs (5 nM). Based on the volumetric analysis of the ionic current blockades shown in Fig. 5, and by invoking similar arguments as presented in Fig. 3, not only we probed the HCV presence at nM concentrations, but we achieved this selectively as AuNP/PNA2–HCV and AuNP/PNA2–H1N1 complexes entailed distinct signatures following reversible interaction with the α-HL. Notably, detection of HCV at similarly low concentrations was precluded in UV–Vis spectra recordings (Supplementary Fig. S6), lending further support of the sensitivity benefit of the presented method.

**Figure 6:**
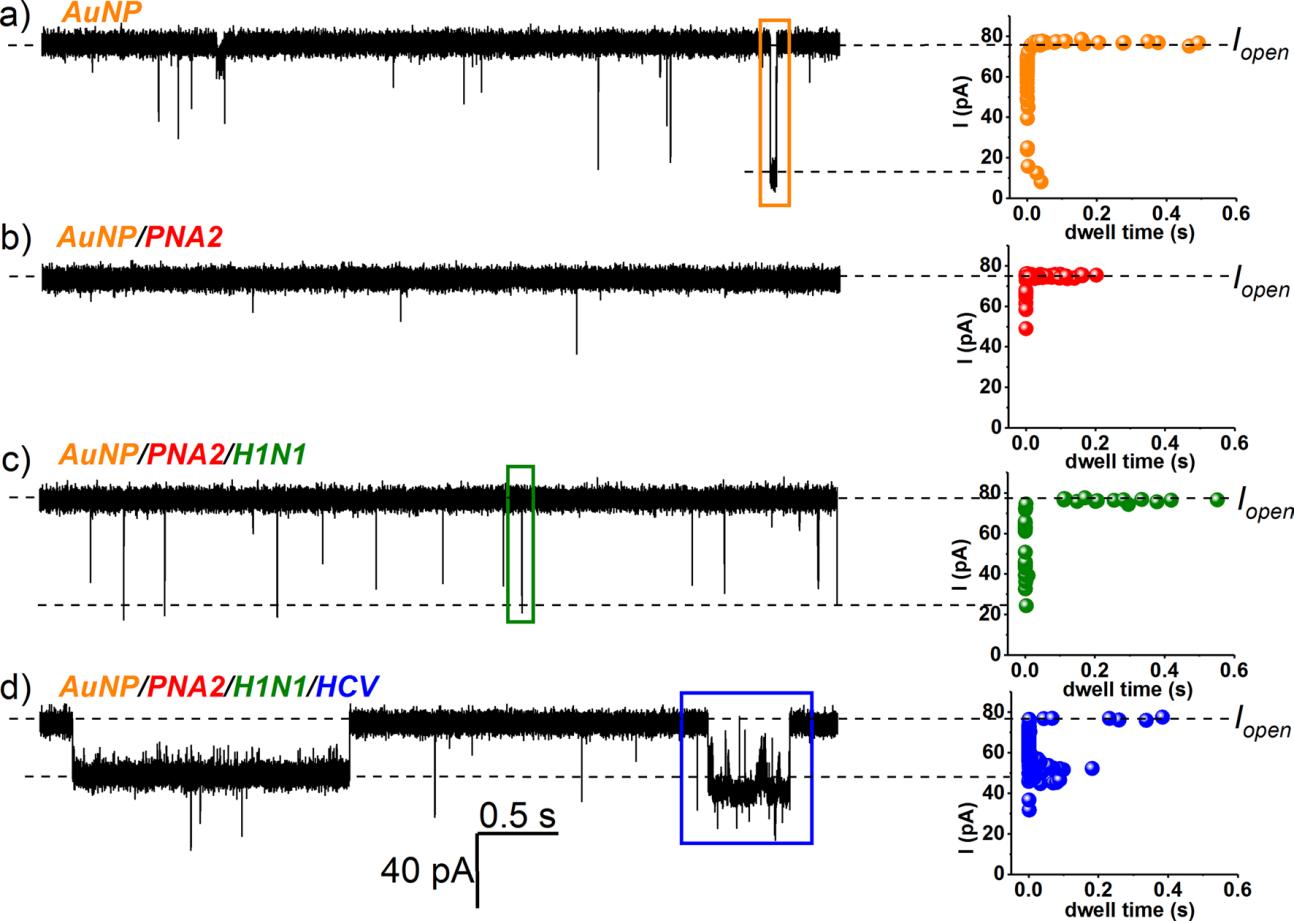
Selective detection of HCV fragments with the AuNP/α-HL platform, in a contaminated sample. Selected recordings (ΔV =  + 70 mV) reflecting *cis*-added AuNP (5 nM)–α-HL interactions (the orange rectangle captures a representative blockade event; ΔI_AuNP_ = 48.1 ± 0.8 pA, τ_off_AuNP_ = 0.001 ± 5E−4 s) (**a**), followed by successive addition of 5 nM PNA2 (**b**), 15 nM H1N1 (the green rectangle captures a representative blockade event; ΔI_H1N1_ = 57.3 ± 0.8 pA, τ_off_H1N1_ = 0.0016 ± 0.001 s) (**c**), and 50 nM HCV (**d**). In the latter case, the appearance of distinct ionic current blockades (i.e., longer in duration and with a smaller amplitude as compared to the AuNP ones, shown representatively in the blue rectangle; ΔI_Au/PNA2/HCV_ = 15.4 ± 0.4 pA, τ_off_Au/PNA2/HCV_ = 0.12 ± 0.01 s), was suggestive of the AuNP/PNA2/HCV complexes and implicit HCV detection.

Next, we revealed that by employing the nanopore technology, the combined use of AuNP and PNA2 can distinguish the individual presence of a target ssDNA species in a mixture. A significant advantage of using PNA fragments for the detection of complementary ssDNA with nanopores, lies in the high hybridization specificity between the PNA and its target ssDNA^66^, rendering the method excellently suited for small nucleic acid fragments sensing in complex mixtures. In representative experiments reflected by Fig. 6, the reagents were added succesively in the *cis* side of nanopore, in the following order: AuNP → PNA2 → H1N1 → HCV. The amplitude analysis of the ensuing blockade events based upon considerations detailed above, demonstrated unequivocally the possibility of detecting the presence of the HCV sequence, and this is highly relevant for applications where selective or multiplex detection of distinct ssDNAs are sought for.

## Conclusions

Herein, we focused on relatively simple, nanopore-based strategy, paving the way for technology development that enables AuNP-assisted, selective detection of short ssDNAs. The assay involved the use of a PNA-induced aggregation of AuNPs, selectively manifested in the presence of target ssDNAs, complementary to the PNA sequence. The subsequent electrophoretic-driven capture of AuNP clusters at the vestibule entrance of a α-HL nanopore isolated in a lipid membrane, and analysis of the ensuing ionic current blockade events, demonstrated a feasible alternative for rapid analysis and sensitive detection of target ssDNAs in a single workflow procedure. The AuNP aggregation strategy reported herein, in conjunction with the use of PNA fragments, paves the way for a rapid, easy, and reliable nucleic acid diagnosis, due mainly to two advantageous attributes: (1) the PNA-DNA hybridization and AuNP aggregation proceed fairly rapid even at room temperature; (2) the number of reagents involved in the assay are reduced to the minimum, precluding the need for expensive primers and enzymes. We also demonstrate the use of the system for detection of target ssDNAs in a mixture. Overall, the developed assay is selective, rapid, and label-free, and appears promising in opening up new avenues in biotechnological and clinical areas. When coupled with a suitable workflow for sample isolation and purification, or other technologies, such as isothermal amplification^83^, or solid-state nanopore systems^38,39,84^, the presented approach could be refined for molecular testing applications in precision medicine involving analysis of free circulating ssDNAs as candidate biomarkers in bodily fluids, in point-of-care units.

## Supplementary information

Supplementary Information.

## Data Availability

All datasets generated during and/or analysed during the current study are available from the corresponding author on reasonable request.
