# Author Correction: Sequence-specific detection of single-stranded DNA with a gold nanoparticle-protein nanopore approach

**DOI:** 10.1038/s41598-020-73154-5

**Published:** 2020-09-30

**Authors:** Loredana Mereuta, Alina Asandei, Isabela S. Dragomir, Ioana C. Bucataru, Jonggwan Park, Chang Ho Seo, Yoonkyung Park, Tudor Luchian

**Affiliations:** 1 Department of Physics, ‘Alexandru I. Cuza’ University, 700506 Iasi, Romania; 2 Sciences Department, Interdisciplinary Research Institute, ‘Alexandru I. Cuza’ University, 700506 Iasi, Romania; 3 Department of Bioinformatics, Kongju National University, Kongju, 32588 Republic of Korea; 4 Department of Biomedical Science and Research Center for Proteinaceous Materials (RCPM), Chosun University, Gwangju, 61452 Republic of Korea

*Correction to*:* Scientific Reports* https://doi.org/10.1038/s41598-020-68258-x, Published online 09 July 2020

This Article contains errors in Supplementary Figure S5, where an incorrect scale is given for the x axis in the inset graphs in panels a) and b). The correct Supplementary Figure S5 appears below as Figure 1.

**Figure 1:**
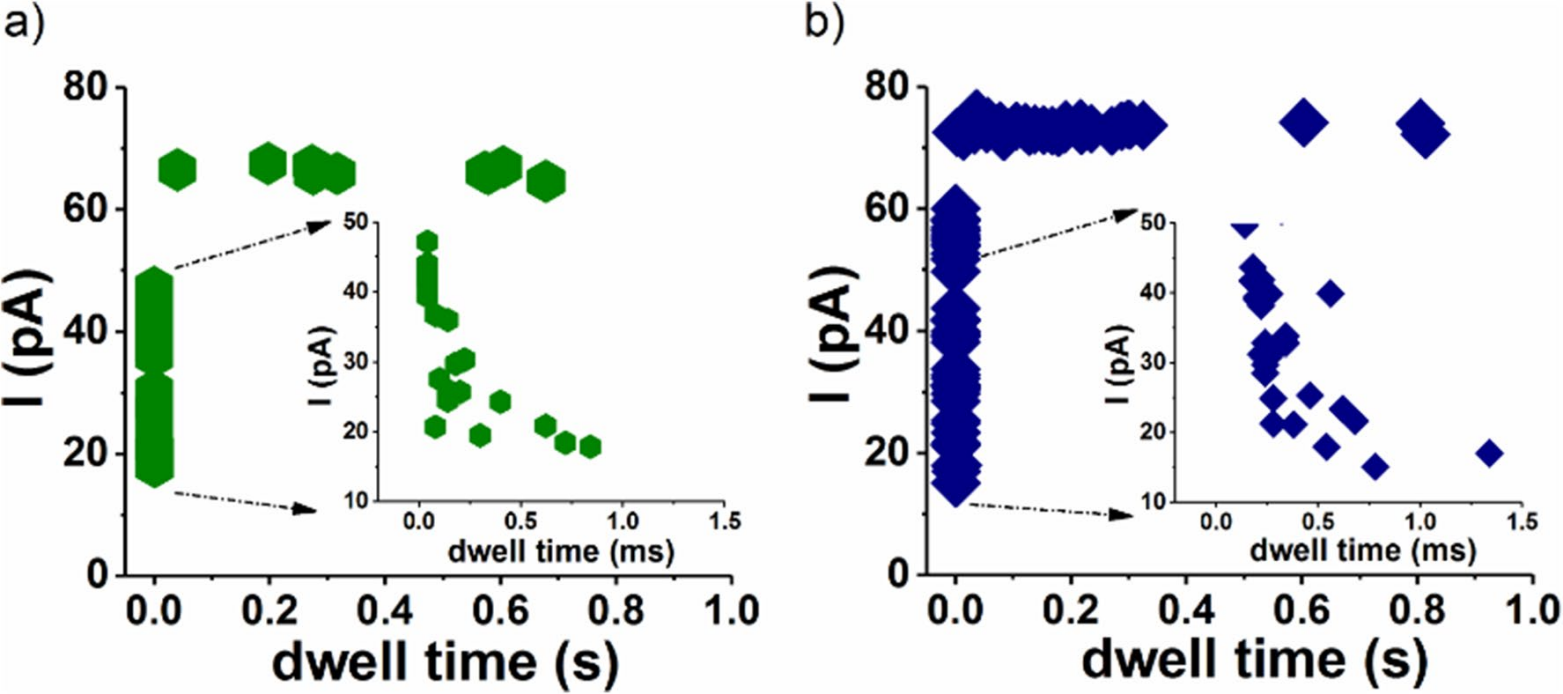
Scatter plot diagrams indicating the ionic current blockade and dissociation dwell time, characterizing H1N1-α-HL (panel a) and HCV-α-HL (panel b) reversible interaction with a single α-HL nanopore, recorded at ΔV =  + 80 mV in asymmetric salt buffers (*trans* 3M KCl / *cis* 0.1M KCl), at neutral pH. ssDNA fragments were added on the *cis* side of the membrane.

