# Supplementary material for: Sequence-specific detection of single-stranded DNA with a gold nanoparticle-protein nanopore approach
Source: Sci Rep. 2020 Jul 9;10:11323. doi: 10.1038/s41598-020-68258-x (PMC7347621; doi:10.1038/s41598-020-68258-x)
Supplement: Supplementary file 1 — Supplementary Information. [file 41598_2020_68258_MOESM1_ESM.docx]

Supplementary Information for:

**Sequence-Specific Detection of Single-Stranded DNA with a Gold**

**Nanoparticle-Protein Nanopore Approach**

Loredana Mereuta^1,#,*^, Alina Asandei^2,#^, Isabela S. Dragomir^2^, Ioana C. Bucataru^1^, Jonggwan Park^3^, Chang Ho Seo^3^, Yoonkyung Park^4,*^, Tudor Luchian^1,*^

^1^ Department of Physics, ‘Alexandru I. Cuza’ University, Iasi, Romania, 700506

^2^ Interdisciplinary Research Institute, Sciences Department, ‘Alexandru I. Cuza’ University, Iasi, Romania, 700506

^3^ Department of Bioinformatics, Kongju National University, Kongju, Republic of Korea, 32588

^4^ Department of Department of Biomedical Science and Research Center for Proteinaceous Materials (RCPM), Chosun University, Gwangju, Republic of Korea, 61452

^#^ These authors contributed equally to this work

^*^ Corresponding authors: [loredana.mereuta@uaic.ro](mailto:loredana.mereuta@uaic.ro) (L.M.); [y_k_park@chosun.ac.kr](mailto:y_k_park@chosun.ac.kr) (Y.P.); [luchian@uaic.ro](mailto:luchian@uaic.ro) (T.L.)





**Figure S1. Spectral UV-vis recordings of citrate-stabilized AuNP (5 nm) in different buffers.** 100 nM AuNP from stock citrate buffer has a surface plasmon peak at ~ 525 nm, which decreases in absolute value but remains visible at the same wavelength when diluted at 10 nM AuNP in distilled water. In a 0.1 M KCl buffer and following up to 30 minutes incubation, there is a slight shift of the surface plasmon peak to ~ 530 nm, indicating the presence of still dispersed AuNPs, whereas in a 3 M KCl buffer we observed a shift of the surface plasmon peak to ~ 580 nm, indicating the AuNPs aggregation.


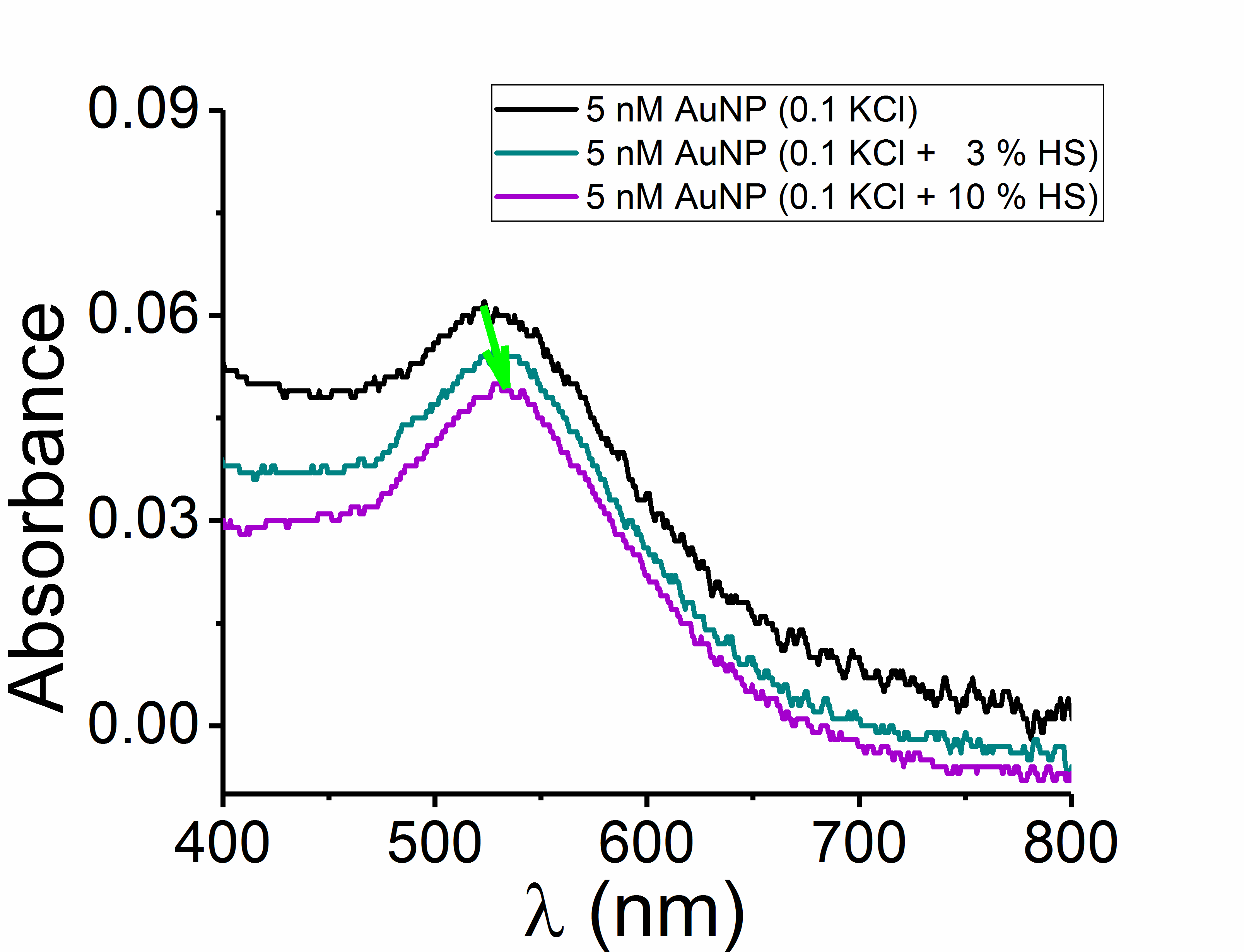


**Figure S2.**  **Effect of human serum on AuNP aggregation**. As compared to the 0.1 M KCl buffer alone, addition of various concentration from human serum (HS) and following 5 minutes incubation has a visible effect on AuNP aggregation, seen as a shift and absolute value decrease of the surface plasmon peak from 528 nm and A_max_=0.06 (no HS, black line) to 532 nm and A_max_=0.055 (3 % HS, green line) and 535 nm and A_max_=0.048 (10 % HS, purple line).

**
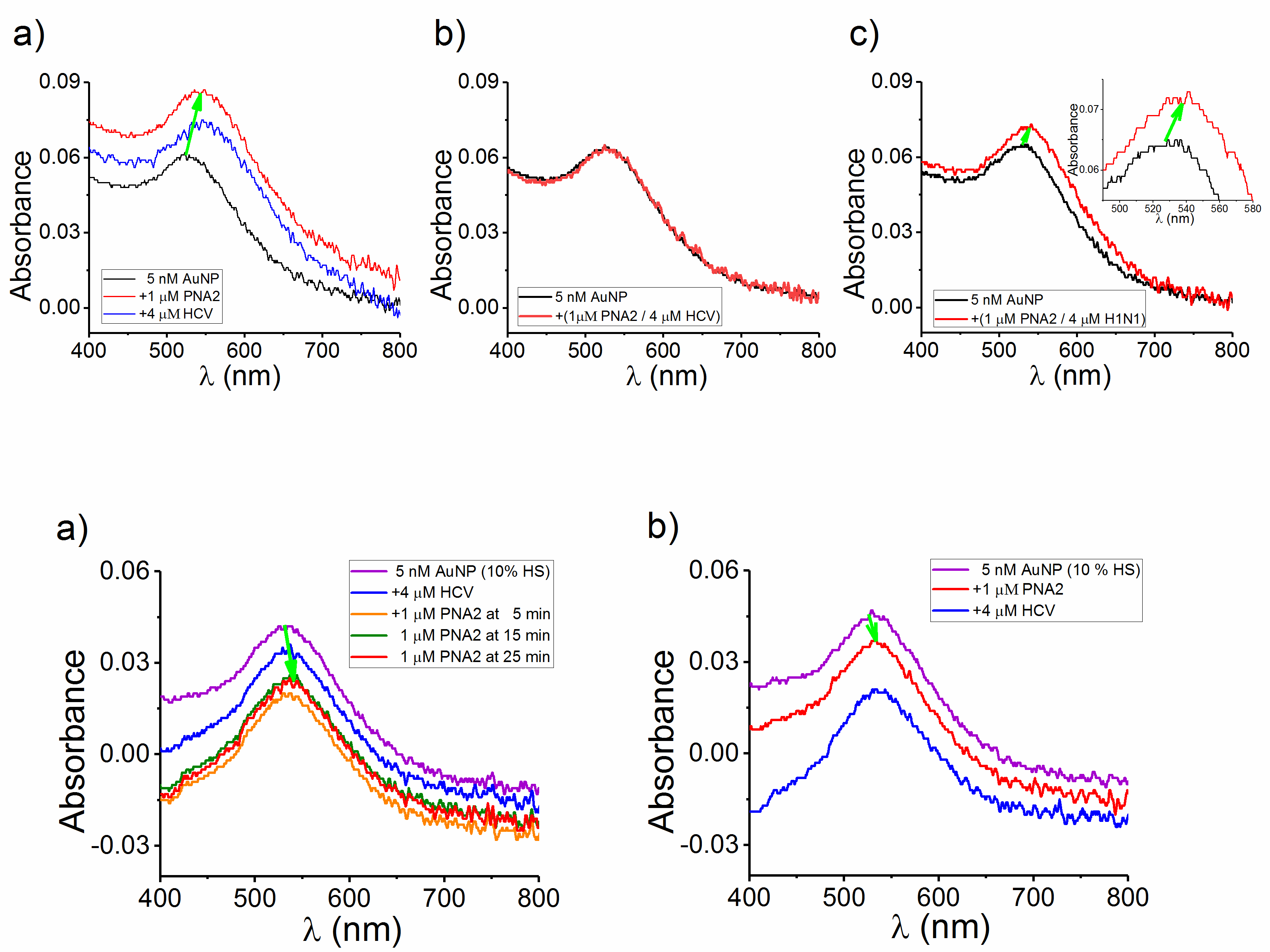
**

**Figure S3.** Spectral UV-vis data recordings on a solution containing 5 nM AuNP dispersed in 0.1 M KCl containing 10% human serum (HS), before and after addition of 4 μM HCV followed by 1 μM PNA2, after different incubation times (a). In panel b we display representative spectra recorded with the order of analytes addition reversed, namely with 1 μM PNA2 present first (the spectrum was measured after 15 minutes incubation time), followed by 4 μM HCV (the spectrum was measured after 25 minutes incubation time). In both panels, the green arrow indicates the ensuing surface plasmon peak shift.

**

**

**Figure S4. Statistical analysis of AuNP-α-HL and HCV-α-HL interactions.** Voltage dependence of the average blockade duration (τ_off_) reflecting the reversible AuNP (5 nM)-α-HL (a) and HCV (4 μM)-α-HL interactions (b).





**Figure S5.** Scatter plot diagrams indicating the ionic current blockade and dissocaition dwell time, characterizing H1N1-α-HL (panel a) and HCV-α-HL (panel b) reversible interaction with a single α-HL nanopore, recorded at ΔV = + 80 mV in asymmetric salt buffers (*trans* 3M KCl / *cis* 0.1 M KCl), at neutral pH. ssDNA fragments were added on the *cis* side of the membrane.

**

**

**Figure S6.** UV-vis spectral data on a 5nM AuNP solution dispersed in 0.1 M KCl, before (surface plasmon peak located at 529 nm and A_max_ = 0.069; black line) and after successive addition of 5 nM PNA2 (surface plasmon peak located at 544 nm and A_max_ = 0.077; red line) and 5 nM HCV (surface plasmon peak located at 544 nm and A_max_ = 0.077; blue line), respectively. The lack of the surface plasmon peak - as evidenced in Fig. 2, for a higher concentration of HCV (main text) - suggests the difficulty in assessing the nanomolar amounts of HVC from spectral analysis on PNA2-HCV complexes - induced aggregation of the AuNP.
